# Consumer Preferences for AI-Based Smart Home Medical Emergency Detection Among German Adults: Choice-Based Conjoint Analysis

**DOI:** 10.2196/94589

**Published:** 2026-09-15

**Authors:** Inga Jagemann, Justin Baudisch, Thorsten Jungeblut, Günter W Maier, Gerrit Hirschfeld

**Affiliations:** 1 Department of Engineering and Mathematics, Hochschule Bielefeld, Bielefeld, North Rhine-Westphalia, Germany, 49 521 106 ext 705; 2 Department of Industrial and Organizational Psychology, Bielefeld University, Bielefeld, North Rhine-Westphalia, Germany; 3 Department of Business, Hochschule Bielefeld, Bielefeld, North Rhine-Westphalia, Germany

**Keywords:** smart home, health monitoring, choice-based conjoint analysis, artificial intelligence, AI literacy

## Abstract

**Background:**

Smart home technology powered by AI can detect anomalies and make emergency calls, enabling residents to live safely and independently. However, the adoption of such technologies for medical emergency detection remains limited.

**Objective:**

This study aimed to explore consumer preferences for AI-based smart home technology for medical emergency detection and identify predictors such as sociodemographic variables, AI literacy, and technology affinity.

**Method:**

A sample of 300 participants (172/300, 57.33% female; 128/300, 42.67% male, aged 18‐69 years) completed a choice-based conjoint analysis (CBCA). Participants evaluated 15 choice sets describing smart home variants based on cost, location, emergency detection rate, type of sensor, and data processing.

**Results:**

Cost was the most important attribute (relative importance [RI]=41%), followed by emergency detection rate (RI=19%), data processing (RI=14%), and location (RI=14%). The type of sensor was the least important attribute (RI=9%). The preferred configuration combined an annual subscription of €70 (US $82), a 95% detection rate, wearable sensors, personalized AI, and installation in both intimate and shared rooms. Notably, 68.3% (205/300) of participants showed a positive none utility, indicating that even the optimal configuration did not overcome general reluctance to adopt such systems. While most expected correlations between sociodemographic variables and attribute importances were not observed, a significant correlation between self-reported health status and emergency detection rate was found (r=.16, *P*=.007). Interestingly, 61% (167/270) of participants preferred AI over human involvement in data processing, but logistic regression revealed that a significant correlation between self-reported health status and emergency detection rate was found (*OR=0.58, P*=.04).

**Conclusions:**

These findings highlight the need to align smart home development with user preferences, emphasizing cost-effectiveness. Additionally, AI literacy plays an important role in technology adoption in the context of AI-based smart home technology. Further research is needed to understand and address the reluctance to adopt AI for medical emergency detection.

## Introduction

AI-based smart home systems incorporate various sensing technologies to monitor resident activities for applications such as health, energy management, and safety. In the context of this study, the term AI refers to machine learning (ML)-based data processing used to recognize patterns in sensor data within the home environment for the detection of medical emergencies, rather than generative AI applications. Particularly in the health domain, these systems are regarded as a promising solution to support older adults and individuals with disabilities living independently at home [1]. For instance, AI can analyze sensor data and detect anomalies to identify patterns indicative of medical emergencies, such as falls or prolonged inactivity, and automatically trigger alerts to emergency services or relatives.

To understand the added value of AI-based smart home systems, it is worth considering the limitations of the technologies they seek to improve upon. They offer advantages over personal emergency response systems (PERSs), which belong to the first generation of home telecare systems and have no embedded intelligence. In case of emergency, the PERS user has to press the help button, worn on a neck chain or as a wristband, which then automatically dials an emergency call center. A study demonstrated that individuals did not use their PERSs to request assistance after a fall [2]. Furthermore, 97% of those who remained on the floor after a fall for over an hour did not use the alarm. Participants attributed this to a lack of perceived benefit, not forming the habit of wearing the PERSs, and difficulties or reluctance in activating the alarm [2]. At the same time, this does not imply that PERSs are generally ineffective, but rather highlights a specific limitation of systems requiring active user intervention in emergency situations. AI-based smart home systems may address this limitation by enabling automated detection without requiring user action. However, their performance depends on the specific technological implementation, and challenges related to sensitivity and specificity remain [3,4].

This potential for medical emergency detection, however, has not translated into widespread adoption, as individuals tend to prefer smart home technology for energy management over health monitoring [5]. While the older population is the primary target demographic for medical emergency detection, their adoption of smart home technology remains relatively low [6,7]. In the United States, 27% of people aged 55 years and older use or own a smart home device (Amazon’s Echo, Nest’s home systems, Google Home, and Samsung’s smart fridges) [8], and in Germany, only 18% of people aged 65 years and older own a smart home device (smart alarm systems, smart lights, and smart hoover) [9].

Despite these potential benefits, the factors underlying low adoption for medical emergency detection remain largely unexplored. From a theoretical perspective, technology acceptance can be understood as a trade-off between perceived usefulness (eg, safety gains), perceived risks (eg, privacy concerns), ease of use, and cost sensitivity, whereby specific system attributes may shape these perceptions differently across users. This raises the question of which attributes of AI-based smart home systems drive acceptance in the context of medical emergency detection.

Therefore, the objective of this study was to use choice-based conjoint analysis (CBCA) to elicit consumer preferences for key attributes of AI-based smart home technologies, including costs, system location, emergency detection rate, sensor type, and data processing. These attributes were selected because they are considered highly relevant, directly influencing the technology’s functionality, usability, and perceived usefulness from the consumer’s perspective. Furthermore, the study aimed to identify sociodemographic variables, technology affinity, and AI literacy as predictors of preferences for the aforementioned attributes. The selected predictors offer insights into the perceptions and engagement of different demographic groups with AI-based smart home systems. These insights could offer developers valuable guidance for designing future products. Specifically, we aim to answer 3 research questions (RQ):

- RQ1: What are the relative importances of the different attributes and part-worth utilities for the different levels considered in the decision-making process for smart home systems?
- RQ2: Does the relative importance of the attributes vary according to sociodemographic factors, technology affinity, and AI literacy?
- RQ3: Does the preference for AI over human involvement in data processing vary according to technology affinity, AI literacy, and sociodemographic factors?

RQ1 aims to describe the preference structure in terms of relative importances of attributes and part-worths of attribute levels and is not associated with specific hypotheses. In the context of the CBCA, relative importances indicate the overall influence of each attribute on participants’ choice decisions, while part-worth utilities represent the estimated preference values associated with the individual levels of each attribute (Table 1). RQ2 is addressed through hypothesis-driven analyses (H2a-f). RQ3 explores differences in preferences for AI versus human data processing without a priori hypotheses.

**Table 1.** Attributes and corresponding levels included in the CBCA^a^.

Attribute	Level
Costs	One-time fee of €1000 (US $1169) One-time fee of €2500 (US $2922) One-time fee of €5000 (US $5843) Subscription €70 per year (US $82)
Type of sensor	Imaging sensors (eg, cameras) Nonimaging sensors (eg, motion detectors and presence detectors) Wearable sensors (eg, smart watch and smart ring)
Emergency detection rate	70% 80% 95%
Location	Intimate rooms (bathroom and bedroom) Shared rooms (living room, kitchen, and floor) Intimate and shared rooms
Data processing	Data processing by AI Data processing personalized AI Data processing by relatives Data processing by care service

a CBCA: choice-based conjoint analysis.

### Relevant Attributes of Smart Home Systems

The attributes for the CBCA were identified based on previous empirical studies on smart home preferences, expert consultation, and the Technology Acceptance Model (TAM; [10]). TAM served as a conceptual backdrop rather than a testable framework: while it identifies perceived usefulness and ease of use as central determinants of acceptance, it leaves the external variables that shape these beliefs unspecified and abstract. We therefore use CBCA and the resulting relative importances to empirically identify which concrete system attributes may function as these external variables. Crucially, CBCA allows us to pit the five attributes against each other simultaneously, revealing which factors most strongly drive adoption decisions—something that examining each attribute in isolation cannot achieve. Rather than replacing TAM, CBCA provides a complementary behavioral perspective by revealing how specific system attributes shape consumer preferences under realistic constraints. The study focused on five key attributes: cost, emergency detection rate, type of sensor, location, and data-processing, summarized in Table 1.

Cost was included as an attribute because it is a key factor in consumer decisions, with high initial prices potentially limiting adoption of emerging technologies [11]. Studies on smart home systems have shown a negative relationship between cost and intention to use [12,13]. To ensure realistic choice scenarios, we based cost levels on actual market prices and included both one-time fees and subscription options. Importantly, the investigated systems were conceptualized as comprehensive smart home emergency detection solutions, potentially involving multiple sensors, whole-home installation, hardware integration, and associated service costs. Accordingly, the selected cost levels were intended to reflect broader smart home infrastructures. While some health care systems may partially reimburse certain emergency response or assistive technologies, reimbursement for comprehensive smart home emergency detection systems is not universally established and may vary considerably across countries and insurance contexts. Therefore, we considered cost to remain a relevant consumer decision factor in the present study.

Emergency detection rate (sensitivity) was included as it represents the primary benefit of smart home technologies for detecting medical emergencies [14,15]. A previous conjoint experiment found that the detection of medical emergencies was the most important attribute for participants [16]. The levels (70%‐95%) were determined in collaboration with experts developing anomaly detection systems, aligning with reported accuracy in activity recognition literature [17].

Type of sensor was included as an attribute because it reflects one of the key advantages of AI-based smart home systems over traditional emergency systems (PERS): automatic, continuous, and context-aware medical emergency detection. In our study, sensors were classified into three groups: wearable, imaging, and nonimaging devices, consistent with existing literature [18]. Nonimaging sensors, which prioritize privacy and energy efficiency, were generally preferred by users over cameras [19-21].

Location of sensors within the home was included as an attribute given the potential trade-off between privacy and safety, as placement in more private spaces such as the bathroom may heighten privacy concerns while simultaneously offering greater safety benefits [22-24]. Individuals are often willing to share personal data if the perceived benefits outweigh the risks [25]. To explore how privacy concerns and perceived usefulness vary across different settings, we analyzed both private rooms (bathroom and bedroom) and shared spaces (living room, kitchen, and floor). Notably, previous research has identified the living room as the preferred location for smart home system installation [19].

Data processing was included as an attribute to examine preferences regarding AI versus human involvement (eg, relatives and caregivers) in evaluating recorded data. Relatives and caregivers refer to human actors responsible for reviewing and interpreting recorded data to identify potential emergencies, in contrast to automated AI-based processing. AI offers several advantages, including constant and automatic availability and reduced burden on family or care services. However, individuals may prefer human oversight due to general risk awareness and AI skepticism in high-stakes health contexts [26,27]. To examine whether framing AI as personalized attenuates this skepticism, we included personalized AI as an additional attribute level, as prior research suggests that personalization has been associated with reduced resistance toward AI and increased trust and adoption intentions [28,29].

### Predictors of Preferences for Specific Design Features

To address RQ2, we formulate a set of hypotheses (H2a-f) examining whether sociodemographic characteristics, technology affinity, and AI literacy systematically predict preferences for specific smart home attributes and data-processing options. Given the limited research, particularly using CBCA on predictors of consumer preferences for smart home technology, this study examines how sociodemographic characteristics (income, technology affinity, living situation, health status, and AI literacy) influence preferences for specific smart home features. Previous questionnaire-based studies have yielded inconclusive results regarding these predictors [30-32], highlighting the need for further investigation. The following section outlines our rationale for the preregistered hypotheses [33].

So far only a handful of studies have explicitly investigated the relationship between income and smart home preferences [30,31,34,35]. While some studies found a relationship between income and intention to use [31,34], another study did not find a relationship between income and user acceptance of smart home services [30]. Arthanat et al [35] found that age, gender, and marital status were not significantly related to smart home ownership, whereas education and income showed a weak, nonsignificant trend toward an association. Based on these results, we expect that income will correlate with the importance of costs (H2a).

Technology affinity, defined as the tendency to actively engage with or avoid technical systems [36], may serve as another predictor. Surveys indicate that higher technology affinity positively affects smart home adoption [19,37,38]. Based on the idea that individuals with lower technology affinity tend to avoid dealing with new technologies as much as possible, we assume that they are especially critical of placing sensors in private rooms. Based on these considerations, we expect that technology affinity will correlate with location preferences within the home (H2b).

Living situation, whether an individual lives alone or with others, may also shape preferences. Research on older adults suggests that those living alone exhibit a higher demand for medical and emergency monitoring [32,39]. However, Yang et al [30] found that the type of residence (single house or multifamily house) was not a significant factor in determining the intention to use smart home services. Based on these results, we expect that participants living alone will correlate with emergency detection rate (H2c).

Health status represents a fourth predictor. Arthanat et al [35] found that physical impairment, falls and accidents, and independence had a significant strong association with smart home ownership. Schuster et al [40] conducted an online survey to explore the correlation between sociodemographic factors and the use of wearable activity trackers and found that self-rated health status was positively correlated with its adoption. We expect that health status will correlate with emergency detection rate (H2d).

Finally, AI literacy, defined as the ability to understand, evaluate, and use AI systems [41], may influence specific design preferences. Previous research shows that greater knowledge of smart home technologies increases perceived benefits and understanding of their potential applications [42,43]. Individuals with higher AI literacy are likely to have a better understanding of how different sensors work and how data are processed, enabling them to make more informed choices regarding sensor types and data-handling options. Based on these considerations, we hypothesize that AI literacy will correlate with preferences for sensor type (H2e) and data-processing (H2f).

## Methods

### Study Design of CBCA

CBCA is a quantitative marketing research method based on the premise that any product or service can be described by its attributes, and that an individual’s valuation of a product or service depends on the levels of these attributes [44]. Respondents are asked to choose between different sets of choices, where each set consists of two or more hypothetical products and their attributes, each with a combination of levels. CBCA offers distinct advantages in the study of consumer preferences, allowing direct comparison of attribute effects and enhancing research realism through choice scenarios, which also help to mitigate social desirability bias [45-48]. The method is increasingly applied to health care settings [49-51] and has great potential for identifying patient preferences to contribute to patient-centered care [52]. Our study is guided by the 10-item checklist for conjoint analysis applications in health care established by the Good Research Practices for Conjoint Analysis Task Force of the International Society for Pharmacoeconomics and Outcomes Research (ISPOR) [53].

**Figure 1.**
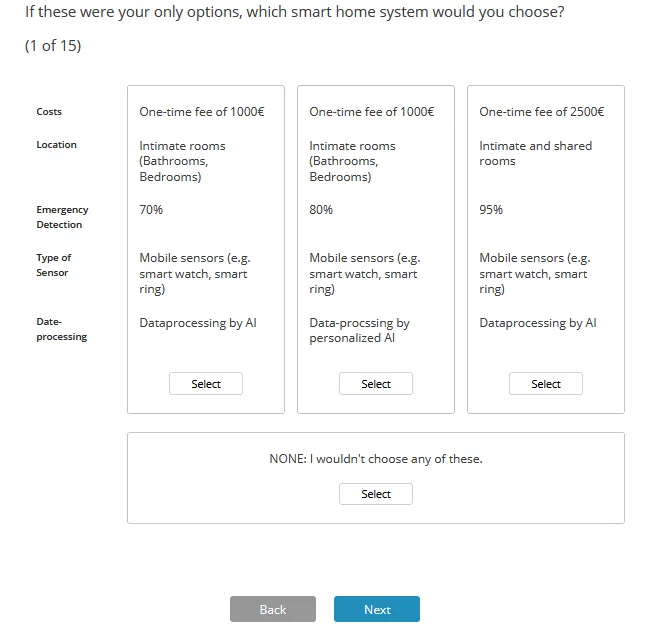
Example choice set (translated from German).

In our study, participants completed a CBCA task consisting of 15 sequential choice sets, each containing three different smart home systems. These choice sets were randomly selected from a total of 432 possible combinations of attribute levels (4×3×3×3×4; Table 1) and were generated using Sawtooth Software’s (Sawtooth Software, Inc.) Balanced Overlap algorithm, which balances level overlap and statistical efficiency [44]. For each set, participants selected their most preferred smart home emergency detection system, designed to detect medical emergencies such as falls or heart attacks, or opted out by choosing none of the presented alternatives. Figure 1 illustrates an example of the choice task. This design allows for the estimation of relative attribute importance and part-worth utilities, providing insights into how users prioritize different features of AI-based smart home systems for medical emergency detection.

### Participants

Recruitment was based on a sample from a market research panel. Data were collected during the time period January 18, 2024, through February 12, 2024. A sample size of 300 participants was determined in accordance with the practical guidelines for conjoint analyses [54,55]. A sensitivity analysis confirmed that this sample size was sufficient to detect a correlation of (*r*=0.2) with 95% power at an α level of .05. The sample was quota-controlled to reflect the distribution of age, gender, and place of residence in the German population. The time exclusion criterion was based on the two-second-per-item rule [56] but was adjusted due to the inclusion of performance-based questions that require more time. Therefore, the criterion was set at 2.68 seconds per item, resulting in a minimum total response time of 180 seconds (3 minutes). To ensure the quality of the responses, we also integrated an attention question into the survey, which resulted in the exclusion of respondents who did not answer correctly.

A total of 455 participants completed the survey; however, 155 of 455 participants (34%) were excluded from the analysis due to their completion time being less than three minutes or their failure to answer the attention check question correctly. The remaining 300 participants were included in the analysis.

### Ethical Considerations

The Psychology Faculty of University of Bielefeld has granted ethical approval (approval number 2023‐325) for this study, and the hypotheses and analyses were preregistered with AsPredicted [33]. The study was conducted in accordance with the ethical standards of the responsible institutional committee on human experimentation and with the World Medical Association Declaration of Helsinki. Prior to participation, all participants received information about the purpose of the study and provided informed consent electronically. At the end of the survey, participants were asked a second time to consent to the use of their data for anonymized analysis. Participation was voluntary, and participants were able to withdraw at any point without providing a reason. Participants were recruited via a market research panel and were compensated according to the panel provider’s standard remuneration scheme.

### Measures

#### Affinity for Technology Interaction Scale

Technology affinity was measured with the unidimensional (German) Affinity for Technology Interaction (ATI) scale, which consists of nine items [36]. A sample item was “When I have a new technical system in front of me, I try it out intensively.” Participants responded on a six-point Likert scale (1=completely disagree to 6=completely agree). The scale was developed with five samples (n>1500). The reliability of the ATI scale was assessed across all samples. Cronbach α values ranged between 0.83 and 0.99 across all samples, indicating good to excellent reliability. The validity analyses demonstrated that ATI shows the expected moderate to strong correlations with related constructs.

#### Objective AI Literacy

Objective AI literacy was measured using a validated AI literacy test [41]. This test consists of 31 single-choice questions with four possible answers for each item, with only one being correct. A sample item was “What makes AI intelligent?” with the four possible answers “AI can walk and talk,” “AI has an artificial brain,” “AI is at least as intelligent as humans,” or “AI acts rationally to achieve a particular goal as well as possible.” The test was developed among German university students (n=1286) and showed high internal reliability (α=.82) and unidimensional structure.

#### Subjective AI Literacy

Furthermore, an additional item was included, which assessed the respondents’ self-rated knowledge about AI. Participants were asked to indicate their level of agreement with the statement “I have a solid understanding of artificial intelligence“ on a six-point Likert scale (1=completely disagree to 6=completely agree).

#### Sociodemographic Characteristics

Additionally, data were collected on a range of sociodemographic characteristics, including age, gender, educational background, academic or vocational training in STEM (science, technology, engineering, and mathematics) subjects, monthly income after tax, health status, usage of emergency services, and living situation. Details of the response options for these variables can be found in Table 2.

**Table 2.** Sample characteristics.

Variables and categories		Values
Sex, n (%)		
Female		172 (57.33)
Male		128 (42.67)
Diverse		0 (0)
Age (years), mean (SD); range		47.22 (13.10); 18‐69
Education, n (%)		
General school leaving certificate (eg, Abitur)		40 (13.33)
Vocational school qualification (eg, apprenticeship)		157 (52.33)
University degree (eg, Bachelor)		92 (30.67)
PhD		5 (1.67)
Other degree		6 (2)
Academic or vocational STEM^a^, n(%)		
Yes		38 (12.67)
No		259 (86.33)
I don’t know		3 (1)
Monthly income after tax (in €), n (%)		
<1000 (<US $1,169)		29 (9.67)
1000-1999 (US $1,169-US $2,336)		76 (25.33)
2000-2999 (US $2,338-US $3,505)		84 (28)
€3000-€3999 (US $3,507-US $4,674)		46 (15.33)
€4000-€4999 (US $4,676-US $5,842)		24 (8)
>€5000 (>US $5,843)		22 (7.33)
Not specified		19 (6.33)
Subjective health status		
Very good		41 (13.67)
Good		154 (51.33)
Average		84 (28)
Bad		20 (6.67)
Very bad		1 (0.33)
Utilization of emergency service, n (%)		
Yes		175 (58.33)
No		123 (41)
Not specified		2 (0.67)
Living situation, n (%)		
Alone		86 (28.67)
With others		214 (71.33)
Subjective AI literacy, mean (SD); range		2.94 (1.24); 1‐6
AI literacy, mean (SD); range		0.41 (0.13); 0.16‐0.81
Technology affinity, mean (SD); range		3.87 (1.01); 1‐6

a STEM: science, technology, engineering, and mathematics

### Procedure

The participants first completed the consent form and agreed to participate in the anonymous study. Subsequently, the participants were provided with a concise overview of the study’s objectives and of the AI-based smart home system, which were defined as the usage of AI-based smart home systems for the detection of emergencies (eg, falls and heart attacks). Following this, the CBCA tasks were provided. The participants then completed the ATI scale [36] and the one-item subjective AI literacy test. After that, the AI literacy choice test was administered [41]. Participants then responded to a series of sociodemographic questions. Finally, respondents were once again requested to grant permission for the usage of their data for anonymized analysis. The average completion time for the survey was 42 minutes.

### Data Analysis

The preregistered data analysis proceeded in three steps. First (RQ1), the relative importances of the attributes and part-worth utilities of the levels were estimated using Hierarchical Bayes (HB) estimation and summarized using standard descriptive statistics. HB estimation was conducted using Sawtooth Software’s Lighthouse Studio with default settings: 10,000 burn-in iterations and 10,000 draws per respondent [57]. A standard HB model without covariates was specified, using effects coding and a target acceptance rate of 0.30. Model fit was satisfactory, with an average root likelihood of 0.596. Logit-based standard errors for all attribute levels ranged from 0.024 to 0.031, well below the recommended threshold of 0.05 [57]. CIs for relative importances and part-worth utilities were estimated using bootstrap resampling, using Hmisc package [58]. Second (RQ2), relations between sociodemographic variables, technology affinity, and AI literacy and the relative importances were explored by using bivariate correlations. Third (RQ3), we used logistic regressions to predict whether and which sociodemographic variables, technology affinity, and AI literacy predicted the preference for AI over human involvement for data processing. The binary dependent variable was constructed from respondents’ individual part-worth utilities for the four data-processing levels. For each respondent, the mean part-worth utility for AI-based processing was computed by averaging the part-worths for AI and personalized AI. Similarly, the mean part-worth utility for human involvement was computed by averaging the part-worths for relatives and care service. Respondents were classified as preferring AI (coded 1) if their mean AI part-worth exceeded their mean human part-worth, and as preferring human involvement (coded 0) otherwise. Prior to analysis, all continuous predictors (AI literacy, technology affinity, subjective AI literacy, age) were divided by two SDs [59]. Generalized variance inflation factors (GVIFs) were computed for all predictors following Fox and Monette [60]. For both logistic regression models, size-adjusted GVIF values were low (RQ3: 1.09‐1.37; exploratory analysis: 1.06‐1.32) and well below the recommended threshold of 5, indicating no problematic multicollinearity [60]. Individual’s part-worth utilities were calculated using Sawtooth Software (version 9.15.0). All other analyses were carried out in R (version 4.3.1; R Foundation for Statistical Computing).

## Results

### Sample

The final sample (N=300) consisted of 172 women (57.33%) and 128 men (42.67%). The mean age of the participants was 47.22 (SD 13.10) years, with a range of 18 to 69 years; notably, no participants aged 70 years or older were included in the sample. The majority of participants had either a vocational school qualification (157/300, 52.33%) or a university degree (92/300, 30.67%), with no vocational or academic training in STEM subjects (259/300, 86.33%). The majority of the participants had a monthly income between €2000 and €2999 (84/300, 28%), described their health status as good (154/300, 51.33%), and lived with others (family, life or marriage partner, flatmates, or others). The characteristics of the sample closely resemble those of the general adult population in Germany [61]. In response to the question of whether they had ever used the services of a hospital’s emergency or medical rescue center due to a medical emergency, the majority of the participants indicated that they had (175/300, 58.33%). Further details can be found in Table 2.

### Relative Importances and Part-Worth Utilities

To address RQ1 regarding the importance of attributes in participants’ preference for smart home systems, presents a summary of the relative importances of the attributes. Overall, costs (41.60%, 95% CI 39.67‐43.53) were the most important attribute, followed by emergency detection rate (19.00%, 95% CI 17.75‐20.25). Data processing (14.90%, 95% CI 13.64‐16.15) and location (14.64%, 95% CI 13.64‐15.65) showed moderate levels of importance, whereas the type of sensor (9.83%, 95% CI 9.10‐10.56) was the least important attribute.

**Figure 2.**
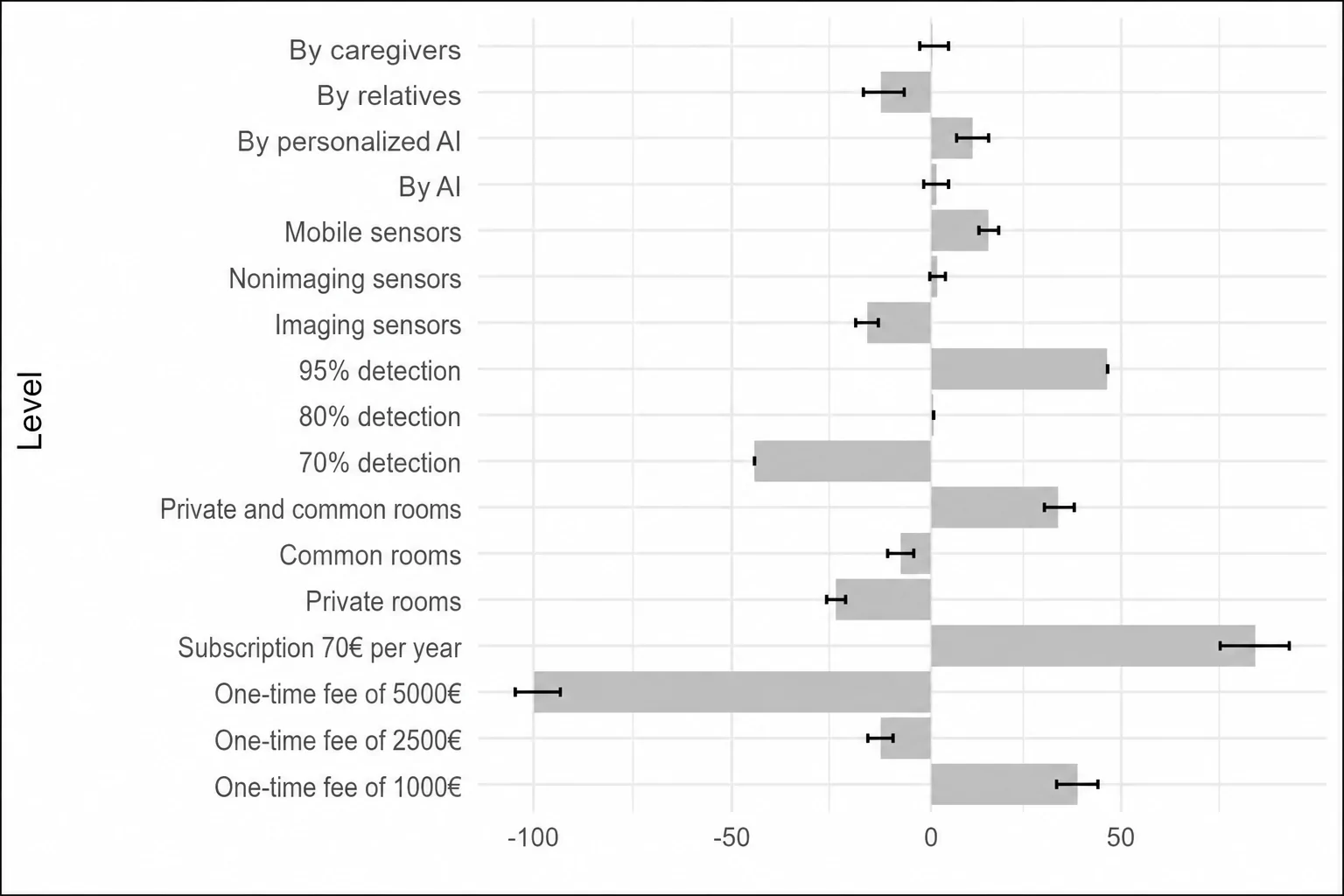
Part-worth utilities indicate strong preferences for subscription-based pricing, a high emergency detection rate (95%), and wearable sensors. Error bars denote 95% CI.

The part-worth utilities for all attributes’ levels are shown in Figure 2. The ideal smart home system (“best case”) costs €70 per year, is installed in private and common rooms, has a detection rate of 95%, uses a wearable sensor, and data are processed by personalized AI. Notably, however, 68.3% (205/300) of participants showed a positive none utility (M=59.56), indicating that even the optimal configuration did not fully overcome participants’ reluctance to adopt a smart home monitoring system. Overall, three aspects are noteworthy. First, participants strongly preferred yearly subscriptions of €70 over one-time fees (76.56, 95% CI 68.28‐84.83). Second, participants demonstrated the strongest preference for data-processing by personalized AI (10.28, 95% CI 6.11‐14.43), followed by AI (1.45, 95% CI −1.82 to 4.73). Third, participants had the strongest preference for wearable sensors (14.38, 95% CI 11.95‐16.80) followed by nonimaging sensors (1.60, 95% CI −0.33 to 3.54).

### Relations Between Sociodemographic Variables and Preferences

To examine research question two regarding the relationship between sociodemographic factors, technology affinity, and AI literacy and the relative importance of attributes, Table 3 provides an overview of the correlations between the variables and the relative importances.

In the next step, we analyzed our preregistered hypotheses (Pearson correlation coefficient). Overall, we found the expected correlations between health status and detection rate (H2d: *r*=0.16, 95% CI 0.04‐0.28*; P*= .007), which indicates that people with a good health status had a higher importance for the attribute detection rate. The association was significant. However, hypotheses H2a, H2b, H2c, H2e, and H2f were not supported (all *P*>.05).

**Table 3.** Pearson correlations between sociodemographic variables and relative importances of smart home system attributes.

Sociodemographic variables	Relative importances (dependent variables), correlation (95% CI)				
	Costs	Detection rate	Sensors	Data processing	Location
Monthly income after tax	0.05 (−0.07 to 0.17)	0.06 (−0.06 to 0.18)	−0.02 (−0.14 to 0.10)	−0.05 (−0.16 to 0.07)	−0.11 (−0.23 to 0.01)
Utilization of emergency service (1: yes, 2: no)	0.05 (−0.07 to 0.17)	−0.04 (−0.16 to 0.08)	−0.07 (−0.19 to 0.05)	0.01 (−0.11 to 0.12)	0.00 (−0.12 to 0.12)
Living-situation (1: alone, 2: with others)	−0.08 (−0.20 to 0.03)	0.12 (0.00 to 0.24)	−0.02 (−0.14 to 0.10)	0.08 (−0.04 to 0.20)	−0.07 (−0.19 to 0.05)
Technology affinity	−0.13^a^ (−0.25 to −0.01)	0.24^b^ (0.12 to 0.35)	−0.15^a^ (−0.26 to −0.03)	0.07 (−0.05 to 0.19)	−0.03 (−0.15 to 0.09)
AI literacy	0.02 (−0.10 to 0.14)	0.03 (−0.09 to 0.15)	−0.05 (−0.17 to 0.07)	−0.11 (−0.23 to 0.01)	0.09 (−0.03 to 0.21)
Subjective AI literacy	−0.04 (−0.16 to 0.07)	0.16^b^ (0.04 to 0.26)	−0.15^b^ (−0.26 to −0.04)	0.07 (−0.04 to 0.18)	−0.09 (−0.20 to 0.02)
Sex (1: female, 2: male)	0.00 (−0.12 to 0.12)	0.25^b^ (0.13 to 0.36)	−0.17^b^ (−0.28 to −0.05)	−0.05 (−0.17 to 0.06)	−0.12^a^ (−0.24 to 0.00)
Age (years)	0.18^b^ (0.06 to 0.29)	−0.23^b^ (−0.34 to −0.11)	0.01 (−0.11 to 0.13)	−0.06 (−0.18 to 0.06)	0.02 (−0.10 to 0.14)
Subjective health status (1: very bad, 5: very good)	−0.07 (−0.19 to 0.05)	0.16^b^ (0.04 to 0.28)	−0.03 (−0.15 to 0.09)	0.03 (−0.09 to 0.15)	−0.09 (−0.21 to 0.03)
Education	0.02 (−0.10 to 0.14)	−0.01 (−0.12 to 0.11)	−0.12^a^ (−0.24 to 0.00)	0.02 (−0.10 to 0.13)	0.03 (−0.09 to 0.15)
Academic or vocational STEM^c^ (1: yes, 2: no)	0.10 (−0.02 to 0.22)	−0.06 (−0.18 to 0.06)	0.01 (−0.11 to 0.13)	−0.11 (−0.23 to 0.01)	0.02 (−0.10 to 0.14)

a *P*<.05.

b *P*<.01.

c STEM: science, technology, engineering, and mathematics.

### Predictors of AI-vs Human Involvement

In order to address research question three, we conducted a logistic regression analysis to determine whether sociodemographic factors, technology affinity, or AI literacy were associated with a preference for AI (AI, personalized AI) over human (relatives, care service) involvement in data-processing. Table 4 presents a summary of the odds for preferences in data-processing. The majority of participants (167/270, 61.85%) expressed a preference for AI in the context of data-processing, while only 38.15% (103/270) preferred human involvement.

**Table 4.** Odds ratios from logistic regression predicting preference for AI over human involvement in data-processing.

Predictors		Preference for AI (1) over human (0), OR^a^ (95% CI)	*P* values	GVIF^b^
Age (years)		1.30 (0.79‐2.14)	.30	1.15
Sex				1.14
Female		—^c^	—	
Male		0.81 (0.49‐1.33)	.41	
Education				1.08
High-school		—	—	
Vocational degree		1.19 (0.57‐2.55)	.64	
University degree or PhD		0.77 (0.35‐1.73)	.52	
Academic or vocational STEM^e^				1.09
No		—	—	
Yes		0.75 (0.34‐1.58)	.46	
Monthly income after tax (in €)				1.06
Less than 1000 (less than US $1,169)		—	—	
1000-1999 (US $1,169–US $2,336)		1.13 (0.45‐2.97)	.80	
2000-2999 (US $2,338–US $3,505)		1.37 (0.56‐3.55)	.50	
3000-3999 (US $3,507–US $4,674)		1.60 (0.60‐4.45)	.35	
4000 or more (US $4,676 or more)		1.00 (0.37‐2.82)	.99	
Utilization of emergency service				1.09
No		—	—	
Yes		0.67 (0.41‐1.10)	.11	
Living situation				1.12
Alone		—	—	
With others		1.15 (0.68‐1.99)	.60	
Health status				1.08
(Very) good		—	—	
Average		1.30 (0.75‐2.23)	.35	
(Very) bad		0.73 (0.25‐1.93)	.55	
AI literacy		0.58 (0.34‐0.97)	.04^d^	1.09
Technology affinity		0.86 (0.53‐1.42)	.56	1.33
Subjective AI literacy		0.78 (0.47‐1.27)	.32	1.33

a OR: odds ratio

b GVIF: size-adjusted generalized variance inflation factor (GVIF1/(2·Df)).

c Not available.

d *P*<.05.

e STEM: science, technology, engineering, and mathematics.

The results indicate that individuals with a high AI literacy (*P*<.05) are less likely to prefer AI in data-processing over human involvement (Table 4). All other variables were found to be nonsignificant (all *P*>.05). Overall, the logistic regression model showed limited predictive utility. Compared to the intercept-only model, the full model did not significantly improve model fit, (*χ*²_16_*=*1613.4; *P*=.64). Pseudo *R*² values were small (McFadden *R²*=0.04; Nagelkerke *R²*=0.07), indicating limited explained variance. The model achieved an AUC of 0.63, suggesting modest discriminatory ability.

To illustrate the practical magnitude of the AI literacy effect, we computed predicted probabilities of preferring AI over human involvement across AI literacy quartiles. The predicted probabilities were 43%, 38%, and 34% at the 25th, 50th, and 75th percentiles of AI literacy, indicating that higher AI literacy is associated with a progressively lower likelihood of preferring AI over human involvement in data processing. To complement the odds ratio with an effect estimate on the probability scale, average marginal effects (AME) were computed. The AME for AI literacy was −0.11 (SE 0.06; *P*=.10), indicating that a two-SD increase in AI literacy is associated with an 11 percentage point decrease in the probability of preferring AI over human involvement, though this effect did not reach conventional significance thresholds.

### Exploratory Analyses

In addition to the preregistered analyses, two further analyses were conducted. The objective of the first analysis was to identify further predictors of individual preferences, while the second analysis explored the status of personalized AI.

Pearson’s correlation coefficient was used to analyze those correlations that were not preregistered. A significant small positive correlation was found between gender and technology affinity (*r*=0.29, 95% CI 0.18‐0.40; *P*<.001), which indicates that male participants have a higher technology affinity. Male participants also have significantly higher AI literacy and significantly higher self-reported AI literacy (Table 5). Furthermore, we found a significant small negative correlation between age and self-reported AI literacy (*r*=−0.29, 95% CI −0.39 to −0.17; *P*<.001), which suggests that younger participants have a higher rating of their knowledge and skills in relation to AI than older participants. Younger participants also have significant higher AI literacy and technology affinity (Table 5). We also found a significant positive correlation between education and AI literacy, which indicated that people with higher education also have a better knowledge about AI (*r*=0.24, 95% CI 0.13‐0.35; *P*<.001). In addition, we found a significant small positive correlation between AI literacy and self-reported AI literacy, suggesting that participants with objectively better knowledge of AI also rate themselves better (*r*=0.12, 95% CI 0.00‐0.24; *P*=.04). Notably, we found a significant strong positive correlation between technology affinity and self-reported AI literacy (*r*=0.61, 95% CI 0.53‐0.68; *P*<.001). Further details can be found in Table 5.

**Table 5.** Pearson correlations between sociodemographic variables, health, technology affinity, and AI literacy.

Variable	Mean (SD)	Gender, correlation (95% CI)	Age, correlation (95% CI)	Education, correlation (95% CI)	Income, correlation (95% CI)	Health status, correlation (95% CI)	Technology affinity, correlation (95% CI)	AI Literacy, correlation (95% CI)	Self-reported AI literacy, correlation (95% CI)
Gender (1=female, 2=male)	1.46 (0.50)	—^a^	−0.36^b^ (−0.46 to –0.25)	0.13^c^ (0.01 to 0.25)	0.20^d^ (0.09 to 0.32)	0.13^c^ (0.01 to 0.25)	0.29^b^ (0.18 to 0.40)	0.18^d^ (0.06 to 0.30)	0.29^b^ (0.18 to 0.39)
Age	46.94 (13.21)	−0.36^b^ (−0.46 to −0.25)	—	−0.06 (−0.18 to 0.06)	−0.06 (−0.18 to 0.06^c^)	−0.28^b^ (−0.38 to −0.16)	−0.26^b^ (−0.37 to –0.15)	−0.12^c^ (−0.24 to −0.01)	−0.29^b^ (−0.39 to −0.17)
Education	2.21 (0.69)	0.13^c^ (0.01 to 0.25)	−0.06 (−0.18 to 0.06)	—	0.27^b^ (0.15 to 0.37)	0.11 (−0.01 to 0.22)	0.13^c^ (0.01 to 0.25)	0.24^b^ (0.13 to –0.35)	0.22^b^ (0.10 to 0.33)
Income	3.11 (1.38)	0.20^d^ (0.09 to 0.32)	−0.06 (−0.18 to 0.06)	0.27^b^ (0.15 to 0.37)	—	0.26^b^ (0.15 to 0.37)	0.17^d^ (0.05 to 0.28)	0.18^d^ (0.06 to 0.29)	0.28^b^ (0.17 to 0.39)
Health status (1=very bad, 5=very good)	3.69 (0.81)	0.13^c^ (0.01 to 0.25)	−0.28^b^ (−0.38 to −0.16)	0.11 (−0.01 to 0.22)	0.26^b^ (0.15 to 0.37)	—	0.19^d^ (0.07 to 0.30)	0.09 (−0.03 to 0.21)	0.23^b^ (0.12 to 0.34)
Technology affinity	3.88 (1.02)	0.29^b^ (0.18 to 0.40)	−0.26^b^ (−0.37 to −0.15)	0.13^c^ (0.01 to 0.25)	0.17^d^ (0.05 to 0.28)	0.19^d^ (0.07 to 0.30)	—	0.15^c^ (0.03 to 0.26)	0.61^b^ (0.53 to 0.68)
AI Literacy	0.42 (0.13)	0.18^d^ (0.06 to 0.30)	−0.12^c^ (−0.24 to −0.01)	0.24^b^ (0.13 to 0.35)	0.18^d^ (0.06 to 0.29)	0.09 (−0.03 to 0.21)	0.15^c^ (0.03 to 0.26)	—	0.12^c^ (0.00 to 0.24)
Self-reported AI literacy	2.94 (1.25)	0.29^b^ (0.18 to 0.39)	−0.29^b^ (−0.39 to −0.17)	0.22^b^ (0.10 to 0.33)	0.28^b^ (0.17 to 0.39)	0.23^b^ (0.12 to 0.34)	0.61^b^ (0.53 to 0.68)	0.12^c^ (0.00 to 0.24)	—

a Not applicable.

b *P*<.001.

c *P*<.05.

d *P*<.01.

In the next step, we analyzed the preferences for personalized AI over AI in data processing. Therefore, we conducted a logistic regression analysis to determine whether sociodemographic factors, technology affinity, or AI literacy were associated with a preference for personalized AI over AI in data processing. The majority of participants (183/270, 67.78%) expressed a preference for personalized AI in the context of data-processing, while only a minority of participants (87/270, 32.22%) indicated a preference for (standard) AI. The results indicate that individuals who live with others (*P*=.02) are more likely to prefer personalized AI over AI for data-processing (Table 6). All other relationships tested were not significant (all *P*>.05). Overall, the model showed modest predictive utility, with acceptable discriminatory ability (AUC=0.67) and small-to-moderate explained variance (McFadden *R²*=0.06; Nagelkerke *R²*=0.11), although the overall model fit was not statistically significant (*χ*²_16_=1621.7; *P*=.15).

**Table 6.** Odds ratios from logistic regression predicting preference for personalized AI over AI in data-processing.

Predictors	Preference for personalized AI (1) over AI (0), OR^a^ (95% CI)	*P* values	GVIF^b^
Age (years)	0.75 (0.45‐1.26)	.28	1.16
Sex			1.15
Female	—^c^	—	
Male	1.41 (0.85‐2.36)	.19	
Education			1.1
High-school	—	—	
Vocational degree	0.97 (0.45‐2.15)	.93	
University degree or PhD	1.02 (0.46‐2.38)	.95	
Academic or vocational STEM^e^			1.1
No	—	—	
Yes	1.56 (0.74‐3.25)	.23	
Monthly income after tax (in €)			1.06
Less than 1000 (less than US $1,169)	—	—	
1000-1999 (US $1,169–US $2,336)	0.56 (0.22‐1.43)	.22	
2000-2999 (US $2,338–US $3,505)	0.58 (0.23‐1.45)	.23	
3000-3999 (US $3,507–US $4,674)	1.16 (0.44‐3.11)	.76	
4000 or more (US $4,676 or more)	0.59 (0.22‐1.62)	.3	
Utilization of emergency service			1.09
No	—	—	
Yes	0.94 (0.56‐1.58)	.81	
Living situation			1.1
Alone	—	—	
With others	2.01 (1.12‐3.75)	.02^d^	
Health status			1.09
(Very) good	—	—	
Average	1.22 (0.70‐2.13)	.48	
(Very) bad	0.37 (0.08‐1.17)	.13	
AI literacy	0.63 (0.36‐1.06)	.09	1.09
Technology affinity	1.46 (0.87‐2.48)	.16	1.29
Subjective AI literacy	1.60 (0.96‐2.71)	.07	1.32

a OR: odds ratio.

b GVIF: size-adjusted generalized variance inflation factor (GVIF1/(2·Df)).

c Not available.

d *P*<.05.

e STEM:science, technology, engineering and mathematics

## Discussion

### Principal Findings

This study examined consumer preferences for AI-based smart home technologies for medical emergency detection and their predictors. With regard to RQ1, cost was by far the most important attribute, followed by emergency detection rate, data-processing, location, and sensor type. The preferred configuration combined subscription-based pricing, a high detection rate, wearable sensors, personalized AI, and installation throughout the entire home. At the same time, the majority of participants showed a positive none utility, indicating substantial residual reluctance to adopt such systems even under optimal conditions. With regard to RQ2, only one of the six preregistered hypotheses was supported: participants reporting better health status placed greater importance on the emergency detection rate, whereas income, living situation, technology affinity, and AI literacy were unrelated to attribute importances. With regard to RQ3, although the majority of participants preferred AI over human involvement in data-processing, higher objective AI literacy was associated with a lower likelihood of this preference. In the following, we discuss these findings in turn before discussing some of the limitations of the present work.

### Comparison With Prior Work

With regard to our first research question, we found that participants had strong and consistent preferences for some of the presented smart home systems. Costs emerged as the most influential attribute, with participants preferring cost-effective subscription-based solutions, consistent with prior research highlighting economic feasibility as a key driver of technology adoption [11,62]. The preference for subscription models can be attributed to the financial flexibility and predictability, allowing users to spread the cost over time and reduce the burden of large upfront investments.

The emergency detection rate was identified as the second most important attribute, with participants strongly favoring systems with the highest detection rate, underscoring the crucial role of safety in medical emergency detection technologies [16,63,64].

The third most relevant attribute was data-processing, which showed a moderate level of importance compared to cost and emergency detection. Participants tended to prefer personalized AI over human (relatives, care service) processing. This preference may reflect a broader trend toward individualization and privacy in digital health technologies [29]. Avoidance of family-based support likely stems from privacy and confidentiality concerns, as users wish to maintain autonomy within their home [65,66].

Location was the fourth important attribute for participants and showed a moderate level of importance. Participants preferred installing smart home systems throughout the entire home, including intimate rooms, which may indicate a desire for comprehensive safety coverage and prioritization of emergency detection. Extending medical emergency detection to private spaces, such as bathrooms where accidents are more likely [67], enhances overall safety and contrasts with previous findings that favored the living room as the optimal installation location [19].

Although sensor type was the least important attribute overall, participants showed a tendency to prefer intelligent wearable sensors over imaging sensors such as cameras, likely due to privacy and intrusiveness concerns. This is consistent with previous research indicating a preference for portable sensors [21] and motion sensors [19]. Intelligent wearable sensors offer flexibility and discretion, allowing for medical emergency detection without the necessity of constant visual surveillance [68]. Participants’ preference for wearable over camera-based sensors further underscores the importance of privacy and nonintrusiveness for technology acceptance.

Based on our own results and previous research, a relatively stable picture emerges. This indicates that potential users prefer wearable smart home solutions that are cost-effective, personalized, installed throughout the entire home, and have a high detection rate. Notably, however, 68.3% (205/300) of participants showed a positive none utility (M=59.56), indicating that even the optimal configuration did not fully overcome participants’ general reluctance to adopt a smart home monitoring system—a finding consistent with the broader adoption gap documented in the literature [6,7]. While the results are unambiguous, it remains unclear how these findings relate to the TAM. Is the preference for AI-based medical emergency detection explained by higher perceived usefulness compared to family-based support? Alternatively, might participants prefer AI-based solutions because relying on family members is perceived as more burdensome?

While this study provides insights into preference structures for AI-based smart home technologies designed to detect medical emergencies, it is important to distinguish these behavior-based stated preferences from actual adoption behavior in real-world settings. The choice tasks in the CBCA require respondents to make explicit trade-offs between system attributes and therefore go beyond purely self-reported attitudes or intentions and have been shown, in some contexts, to predict actual behavior [45,69]. At the same time, these choices in hypothetical scenarios cannot fully capture the broader set of contextual and structural constraints that may affect real-world adoption, such as installation requirements, maintenance effort, trust in service providers, regulatory frameworks, or existing institutional care arrangements. Consequently, the findings should be interpreted as reflecting relative preference patterns under controlled choice conditions, rather than as direct predictors of actual system uptake.

Regarding RQ2, most expected correlations between predictors and attribute importance were not observed. As anticipated, individuals with better health status placed greater emphasis on emergency detection rates, consistent with previous findings linking physical impairments, falls, and accidents to smart home adoption, as well as self-rated health to wearable activity tracker use [35,40].

No significant associations were found for income, living situation, technology affinity, or AI literacy. While previous studies have reported effects of income on smart home adoption and purchase intentions [31,34,35] as well as associations between living situation and smart home usage intentions [39], our findings align partly with Yang et al [30], who also reported no significant effects for income and housing-related variables. Similarly, although prior research identified technology affinity as a predictor of general smart home adoption [19,37,38] and AI knowledge as shaping perceived benefits and use cases of smart home technologies [42,43], these effects were not observed for the specific attribute preferences examined here. These discrepancies may reflect methodological differences, as prior studies primarily assessed general acceptance or usage intentions, whereas this study focused on concrete trade-offs between specific system attributes.

Regarding our RQ3, we found that a greater proportion of participants preferred to have their data processed by AI rather than by humans. At the same time, the logistic regression analysis showed that individuals with higher objective AI literacy were less likely to prefer AI-based data-processing over human involvement. Predicted probabilities further illustrated this effect by revealing a gradual decline in AI preference with increasing AI literacy, while simultaneously indicating that AI-based data-processing remained a viable option for a substantial proportion of participants across all literacy levels. One possible explanation is that individuals with higher AI literacy may be more aware of potential drawbacks of AI systems, including technical failures, misinterpretations leading to missed or false emergency alerts [70], and data security or privacy risks [71]. Beyond heightened risk awareness, greater AI literacy may also be associated with increased skepticism regarding the reliability of AI systems in high-stakes health contexts, stronger preferences for accountability and human oversight, or concerns about opaque decision-making processes. In addition, individuals with higher AI literacy may place greater trust in established institutions or human caregivers than in fully automated systems. We would like to explicitly distance ourselves from the notion that AI literacy should be reduced in order to increase the preference for AI-based systems. Rather, the findings suggest that individuals with lower objective AI literacy may overestimate the benefits of AI-based smart home technologies, potentially due to simplified mental models of AI functionality. This interpretation is consistent with TAM, in which perceived usefulness—rather than actual technical understanding—plays a central role in shaping usage intentions. Accordingly, perceptions of the usefulness of AI-based smart home technology may influence preferences for AI-driven data processing even when objective understanding is limited.

Our first exploratory analysis showed that younger participants, males, and those with higher education exhibited higher levels of technology affinity, AI literacy, and self-reported AI literacy. This is in line with the study by Franke et al [36], who found that men have a significantly higher ATI than women. They also found a significant weak negative correlation between age and ATI. However, they did not find significant relationships between educational background and ATI values [36]. Another study has shown that older adults with the lowest levels of education possess the least knowledge about AI [72]. The observed correlation between objective and subjective AI literacy was low, whereas subjective AI literacy showed a stronger correlation with technology affinity.

Our second exploratory analysis showed that the majority of participants expressed a preference for personalized AI in the context of data processing, while only a minority of participants indicated a preference for (standard) AI. The incorporation of personalization into AI technology has the potential to address concerns related to privacy and trust, thereby facilitating greater acceptance and adoption of AI-driven solutions, which is in accordance with previous research [28]. The analysis also indicates that individuals who live with others are more likely to prefer personalized AI over AI for data processing. The inclination toward personalized solutions may suggest that individuals residing in shared households have a heightened expectation of the adaptability of technology. In a shared household, smart home technologies must be more flexible and adaptable to the varying preferences and requirements of the individuals residing there.

### Implications

The findings of this study yield significant implications for product development and policy-making. Practically, product designers of AI-based smart home health systems should offer flexible, subscription-based pricing to reduce upfront costs and ease market entry, while emphasizing enhanced safety through reliable emergency detection, user autonomy through personalized AI-based data processing, and nonintrusive whole-home sensing technologies in both product design and marketing. The observed inverse relationship between objective AI literacy and the preference for AI-driven data processing underscores that perceptions of usefulness, rather than actual technical knowledge, are key drivers of acceptance. For individuals with high objective AI literacy, who tend to be more cautious and less likely to prefer smart home systems, designers should focus on building trust by providing detailed yet clear explanations of data-processing, potential errors, and privacy safeguards, along with transparent demonstrations of how risks are mitigated. For those with lower AI literacy, who may overestimate the benefits and show higher acceptance, designers should aim to set realistic expectations, highlighting both capabilities and limitations of AI while reinforcing tangible benefits, and offering hands-on experiences that make the system’s value immediately understandable. On a theoretical level, CBCA serves as a complement to TAM rather than a replacement. While TAM identifies perceived usefulness and ease of use as central determinants of acceptance, it leaves unspecified which concrete system attributes shape these beliefs. CBCA addresses this gap by empirically identifying which attributes most strongly drive adoption decisions, thereby translating TAM’s abstract constructs into observable consumer preferences under realistic constraints.

### Limitations

The interpretation of this study needs to take several limitations into account. First, while the applied speed and attention criteria were necessary to ensure data quality, the exclusion of 34% of completers may have introduced selection bias. In particular, such criteria may disproportionately exclude individuals with lower literacy, lower motivation, or reduced familiarity with survey formats—groups that may face greater barriers to adopting AI-based smart home health technologies. Consequently, the observed preference structures may somewhat overrepresent more attentive and technologically engaged respondents. Second, as a cross-sectional study, this research is unable to distinguish between age, period, and cohort effects, which may have contributed to the observed results [73]. Third, although AI-based smart home systems designed to detect medical emergencies are often considered particularly beneficial for older adults, the age distribution of this sample was skewed toward middle-aged participants. Since no participants aged 70 years or older were included, the findings may not fully reflect the preferences and needs of older population segments. Fourth, system performance was operationalized solely via emergency detection rate (ie, sensitivity or true positive rate), while specificity (ie, the rate of correctly identifying nonemergency situations and avoiding false alarms) was not explicitly considered. Future research should examine how trade-offs between detection sensitivity and specificity influence consumer preferences. Fifth, the AI literacy measure [41] was validated on university students and, at the time of data collection, represented the only available German-language performance-based measure. In this sample, six of 31 items were solved correctly by fewer than 30% of participants, indicating floor effects that likely compressed variance and may have attenuated the associations hypothesized for H2e and H2f. However, recalculating the correlations for H2e and H2f using a reduced AI literacy score that excluded these six low-performing items yielded substantively unchanged results, with both associations remaining nonsignificant. Sixth, the relative importance of the attributes under investigation is contingent on the specific set of attributes included in the conjoint design. To facilitate comparability across studies, future research may benefit from using common anchor attributes, such as pricing information. Finally, the sample size of this study was insufficient for applying advanced segmentation techniques such as cluster analysis. Nevertheless, a sensitivity analysis demonstrated that the sample size was sufficient to detect a correlation of *r*=0.20 with a power of 95% at an α level of .05.

## Conclusion

Users evaluate AI-based smart home systems through a pragmatic lens, in which cost, reliability, and privacy outweigh technical implementation details such as the specific sensor technology. Beyond guiding product design, this pattern raises a broader question about how such systems reach those who need them: if affordability is the dominant barrier, individual design optimization alone will not close the adoption gap, and reimbursement schemes and structural integration into existing care arrangements deserve greater attention than they have received so far. This is reinforced by the substantial residual reluctance we observed even for the optimal configuration, which suggests that acceptance is shaped by factors extending beyond the attributes of the system itself. At the same time, the lack of strong effects for most sociodemographic variables points to a relatively broad consensus regarding what users expect from such systems. The differences that did appear, such as the link between higher objective AI literacy and a reduced preference for purely AI-based data processing, highlight a nuanced relationship between knowledge and trust: familiarity with AI does not necessarily translate into uncritical endorsement. Moreover, while such technologies can automate tasks and ease caregiver burden, AI-based smart home health systems cannot replace human empathy and social interaction, meaning their use must always consider residents’ mental as well as physical well-being.

## Abbreviations

AME: average marginal effects
ATI: Affinity for Technology Interaction Scale
CBCA: choice-based conjoint analysis
GVIF: Generalized Variance Inflation Factor
HB: Hierarchical Bayes
ISPOR: International Society for Pharmacoeconomics and Outcomes Research
ML: machine learning
PERS: personal emergency response systems
RI: relative importance
RQ: research questions
STEM: science, technology, engineering, and mathematics
TAM: Technology Acceptance Model

## References

[1] Morita PP, Sahu KS, Oetomo A (2023). Health monitoring using smart home technologies: scoping review. JMIR Mhealth Uhealth.

[2] Fleming J, Brayne C, Cambridge City over-75s Cohort (CC75C) study collaboration (2008). Inability to get up after falling, subsequent time on floor, and summoning help: prospective cohort study in people over 90. BMJ.

[3] Gattani A, Dixit S, Patil M (2026). Artificial intelligence for fall detection in older adults: a comprehensive survey of machine learning, deep learning approaches, and future directions. Ageing Res Rev.

[4] Maray N, Ngu AH, Ni J, Debnath M, Wang L (2023). Transfer learning on small datasets for improved fall detection. Sensors (Basel).

[5] Schomakers EM, Biermann H, Ziefle M (2021). Users’ preferences for smart home automation – investigating aspects of privacy and trust. Telemat Inform.

[6] Wei W, Gong X, Li J, Tian K, Xing K (2023). A study on community older people’s willingness to use smart home-an extended technology acceptance model with intergenerational relationships. Front Public Health.

[7] Arthanat S, Wilcox J, Macuch M (2019). Profiles and predictors of smart home technology adoption by older adults. OTJR (Thorofare N J).

[8] Awareness of a smart or connected home device in the United States in 2022, by age 2022. Statista.

[9] (2022). Das intelligente zuhause. Bitkom.

[10] Davis FD (1989). Perceived usefulness, perceived ease of use, and user acceptance of information technology. MIS Q.

[11] Yusif S, Soar J, Hafeez-Baig A (2016). Older people, assistive technologies, and the barriers to adoption: a systematic review. Int J Med Inform.

[12] Al-Husamiyah A, Al-Bashayreh M (2022). A comprehensive acceptance model for smart home services. Int J Data Netw Sci.

[13] Park E, Cho Y, Han J, Kwon SJ (2017). Comprehensive approaches to user acceptance of Internet of Things in a smart home environment. IEEE Internet Things J.

[14] Majumder S, Aghayi E, Noferesti M (2017). Smart homes for elderly healthcare—recent advances and research challenges. Sensors (Basel).

[15] Percy Campbell J, Buchan J, Chu CH, Bianchi A, Hoey J, Khan SS (2024). User perception of smart home surveillance among adults aged 50 years and older: scoping review. JMIR Mhealth Uhealth.

[16] Arning K, Ziefle M, Geissbühler A, Demongeot J, Mokhtari M, Abdulrazak B, Aloulou H (2015). Lecture Notes in Computer Science.

[17] Dahmen J, Cook DJ (2021). Indirectly-supervised anomaly detection of clinically-meaningful health events from smart home data. ACM Trans Intell Syst Technol.

[18] Mokhtari G, Aminikhanghahi S, Zhang Q, Cook DJ (2018). Fall detection in smart home environments using UWB sensors and unsupervised change detection. J Reliab Intell Environ.

[19] Arar M, Jung C, Awad J, Chohan AH (2021). Analysis of smart home technology acceptance and preference for elderly in Dubai, UAE. Designs.

[20] Paudel R, Eberle W, Holder L Anomaly detection of elderly patient activities in smart homes using a graph-based approach. https://eecs.wsu.edu/~holder/pubs/ICD8019.pdf.

[21] Sitar-Taut AV, Sitar-Taut DA, Cramariuc O (2018). Smart homes for older people involved in rehabilitation activities - reality or dream, acceptance or rejection?. Balneo.

[22] Soppera A, Burbridge T, Sammes AJ, Steventon A, Wright S (2006). Intelligent Spaces.

[23] Hoy MB (2018). Alexa, Siri, Cortana, and more: an introduction to voice assistants. Med Ref Serv Q.

[24] Orlowski A, Loh W (2025). Data autonomy and privacy in the smart home: the case for a privacy smart home meta-assistant. AI & Soc.

[25] Maier E, Doerk M, Reimer U, Baldauf M (2023). Digital natives aren’t concerned much about privacy, or are they?. I Com (Berl).

[26] Castelo N, Bos MW, Lehmann DR (2019). Task-dependent algorithm aversion. J Mark Res.

[27] Bigman YE, Gray K (2018). People are averse to machines making moral decisions. Cognition.

[28] Longoni C, Bonezzi A, Morewedge CK (2019). Resistance to medical artificial intelligence. J Consum Res.

[29] Komiak SYX, Benbasat I (2006). The effects of personalization and familiarity on trust and adoption of recommendation Agents1. MIS Q.

[30] Yang H, Lee H, Zo H (2017). User acceptance of smart home services: an extension of the theory of planned behavior. Ind Manage Data Syst.

[31] Shin J, Park Y, Lee D (2018). Who will be smart home users? An analysis of adoption and diffusion of smart homes. Technol Forecast Soc Change.

[32] Kong D, Liu S, Hong Y, Chen K, Luo Y (2023). Perspectives on the popularization of smart senior care to meet the demands of older adults living alone in communities of Southwest China: a qualitative study. Front Public Health.

[33] (2024). Consumer preferences for smart-home sensors and data-processing. AsPredicted.

[34] Chang S, Nam K (2021). Smart home adoption: the impact of user characteristics and differences in perception of benefits. Buildings.

[35] Arthanat S, Chang H, Wilcox J (2020). Determinants of information communication and smart home automation technology adoption for aging-in-place. J Enabling Technol.

[36] Franke T, Attig C, Wessel D (2019). A personal resource for technology interaction: development and validation of the Affinity for Technology Interaction (ATI) Scale. Int J Hum Comput Interact.

[37] Hubert M, Blut M, Brock C, Zhang RW, Koch V, Riedl R (2019). The influence of acceptance and adoption drivers on smart home usage. Eur J Mark.

[38] Iten R, Wagner J, Zeier Röschmann A (2024). On the adoption of smart home technology in Switzerland: results from a survey study focusing on prevention and active healthy aging aspects. Smart Cities.

[39] Tural E, Lu D, Austin Cole D (2021). Safely and actively aging in place: older adults’ attitudes and intentions toward smart home technologies. Gerontol Geriatr Med.

[40] Schuster AM, Kadylak T, Cotten SR (2023). Correlation between socio-demographic factors and adoption and use of wearable activity trackers in online American older adults. Educ Gerontol.

[41] Hornberger M, Bewersdorff A, Nerdel C (2023). What do university students know about artificial intelligence? Development and validation of an AI literacy test. Comput Educ Artif Intell.

[42] Sovacool BK, Martiskainen M, Furszyfer Del Rio DD (2021). Knowledge, energy sustainability, and vulnerability in the demographics of smart home technology diffusion. Energy Policy.

[43] Shank DB, Wright D, Lulham R, Thurgood C (2021). Knowledge, perceived benefits, adoption, and use of smart home products. Int J Hum Comput Interact.

[44] Chrzan K, Orme B (2000). An overview and comparison of design strategies for choice-based conjoint analysis. SawTooth Software Research Paper Series.

[45] Hainmueller J, Hangartner D, Yamamoto T (2015). Validating vignette and conjoint survey experiments against real-world behavior. Proc Natl Acad Sci USA.

[46] Hainmueller J, Hopkins DJ, Yamamoto T (2014). Causal inference in conjoint analysis: understanding multidimensional choices via stated preference experiments. Polit Anal.

[47] Knudsen E, Johannesson MP (2019). Beyond the limits of survey experiments: how conjoint designs advance causal inference in political communication research. Polit Commun.

[48] Horiuchi Y, Markovich Z, Yamamoto T (2022). Does conjoint analysis mitigate social desirability bias?. Polit Anal.

[49] Marshall D, Bridges JFP, Hauber B (2010). Conjoint analysis applications in health - how are studies being designed and reported?: An update on current practice in the published literature between 2005 and 2008. Patient.

[50] Larsen A, Tele A, Kumar M (2021). Mental health service preferences of patients and providers: a scoping review of conjoint analysis and discrete choice experiments from global public health literature over the last 20 years (1999-2019). BMC Health Serv Res.

[51] Kim M, Oh J, Kim D, Shin J, Lee D (2025). Understanding user preferences in developing a mental healthcare AI chatbot: a conjoint analysis approach. Int J Hum Comput Interact.

[52] Ryan M, Farrar S (2000). Using conjoint analysis to elicit preferences for health care. BMJ.

[53] Bridges JFP, Hauber AB, Marshall D (2011). Conjoint analysis applications in health--a checklist: a report of the ISPOR good research practices for conjoint analysis task force. Value Health.

[54] Halversen C (2020). Sample size rule of thumb for choice-based conjoint (CBC). Sawtooth.

[55] Orme B (2019). Sample size issues for conjoint analysis. Sawtooth Software.

[56] Bowling NA, Huang JL, Brower CK, Bragg CB (2023). The quick and the careless: the construct validity of page time as a measure of insufficient effort responding to surveys. Organ Res Methods.

[57] (2024). HB estimation settings. Sawtooth Software.

[58] Harrell Jr FE (2026). Hmisc: Harrell miscellaneous. r package version 5.2-6. Comprehensive R Archive Network (CRAN).

[59] Gelman A (2008). Scaling regression inputs by dividing by two standard deviations. Stat Med.

[60] Fox J, Monette G (1992). Generalized collinearity diagnostics. J Am Stat Assoc.

[61] (2022). Mikrozensus- Haushalte Und Familien: Endergebnisse.

[62] Pirzada P, Wilde A, Doherty GH, Harris-Birtill D (2022). Ethics and acceptance of smart homes for older adults. Inform Health Soc Care.

[63] European Commission (2023). Population structure and aging.

[64] Roquebert Q, Sicsic J, Rapp T, SPRINT-T Consortium (2021). Health measures and long-term care use in the European frail population. Eur J Health Econ.

[65] Berridge C, Wetle TF (2020). Why older adults and their children disagree about in-home surveillance technology, sensors, and tracking. Gerontologist.

[66] Slane A, Pedersen I (2025). Bringing older people’s perspectives on consumer socially assistive robots into debates about the future of privacy protection and AI governance. AI & Soc.

[67] Schellenberg M, Inaba K, Chen J (2019). Falls in the bathroom: a mechanism of injury for all ages. J Surg Res.

[68] Amato F, Balzano W, Cozzolino G (2022). Design of a wearable healthcare emergency detection device for elder persons. Appl Sci.

[69] Quaife M, Terris-Prestholt F, Di Tanna GL, Vickerman P (2018). How well do discrete choice experiments predict health choices? A systematic review and meta-analysis of external validity. Eur J Health Econ.

[70] Ghorayeb A, Comber R, Gooberman-Hill R (2021). Older adults’ perspectives of smart home technology: are we developing the technology that older people want?. Int J Hum Comput Stud.

[71] Horowitz MC, Kahn L, Macdonald J, Schneider J (2023). Adopting AI: how familiarity breeds both trust and contempt. AI Soc.

[72] Wang X, Ellul J, Azzopardi G (2020). Elderly fall detection systems: a literature survey. Front Robot AI.

[73] Bell A (2020). Age period cohort analysis: a review of what we should and shouldn’t do. Ann Hum Biol.

[74] Jagemann I, Baudisch J, Jungeblut T, Maier G, Hirschfeld G (2024). Dataset for: the more you know, the less you want to rely on it –consumer preferences for AI-based health monitoring in smart home systems. Zenodo.

